# *Clonorchis sinensis* Crude Antigen Suppresses Osteoclast Differentiation via Modulation of the NF‐κB and MAPK Signaling Pathway

**DOI:** 10.1002/iid3.70292

**Published:** 2025-11-05

**Authors:** Lili Tang, Shanshan He, Buguo Ma, Zeli Tang, Tingzheng Zhan, Suyu Xiao, Jilong Wang, Jinmin Zhao, Jiake Xu, Yunliang Shi

**Affiliations:** ^1^ Parasitology Department, School of Basic Medical Sciences Guangxi Medical University Nanning China; ^2^ Guangxi Key Laboratory of Regenerative Medicine Guangxi Medical University Nanning Guangxi China; ^3^ Education Department of Guangxi Zhuang Autonomous Region Key Laboratory of Basic Research on Regional Diseases (Guangxi Medical University) Nanning China; ^4^ Collaborative Innovation Centre of Regenerative Medicine and Medical BioResource Development and Application Co‐constructed by the Province and Ministry Guangxi Medical University Nanning Guangxi China; ^5^ Histology and Embryology Department, School of Basic Medical Sciences Guangxi Medical University Nanning China; ^6^ Cell Biology and Genetics Department, School of Basic Medical Sciences Guangxi Medical University Nanning China; ^7^ Faculty of Pharmaceutical Sciences Shenzhen University of Advanced Technology, Chinese Academy of Sciences Shenzhen China; ^8^ School of Biomedical Sciences The University of Western Australia Perth Western Australia Australia

**Keywords:** *Clonorchis sinensis* crude antigen, MAPK, NF‐κB, osteoclast differentiation, osteoimmunology

## Abstract

**Background:**

*Clonorchis sinensis* crude antigen (*Cs*CA) is recognized for its immunomodulatory capacity in host‐pathogen interactions and immune‐related pathologies, its direct impact on bone remodeling cells remains underexplored. Although prior studies demonstrate *Cs*CA‐mediated suppression of inflammatory bone formation in ankylosing spondylitis, the specific regulatory effects on osteoclastogenesis and associated molecular mechanisms remain elusive. This study aimed to investigate the impact of *Cs*CA on osteoclast differentiation and its potential molecular mechanisms.

**Methods:**

The cytotoxicity of *Cs*CA on mouse bone marrow–derived macrophages (BMMs) was evaluated using the CCK‐8 assay. At the established non‐cytotoxic dose, the effects of *Cs*CA on osteoclast morphology and activity were observed via TRAcP staining and F‐actin ring formation assay. Expression of osteoclast differentiation‐related genes and proteins was analyzed by combined RT‐qPCR and Western Blot. RNA‐seq was performed to identify *Cs*CA‐regulated signaling pathways during osteoclast differentiation, followed by validation of key pathway components through Western Blot and immunofluorescence.

**Results:**

*Cs*CA exhibited no significant cytotoxicity on BMMs at concentrations ≤ 40 μg/mL. TRAcP staining revealed a significant reduction in osteoclast numbers in *Cs*CA‐treated groups. F‐actin ring formation assays demonstrated abnormal cytoskeletal structures. RT‐PCR results showed significantly downregulated expression of osteoclast differentiation‐related genes, including *NFATc1*, *c‐Fos*, *Acp5*, *CTSK* and *MMP‐9*. Western Blot confirmed reduced protein expression levels of NFATc1 and c‐Fos. RNA‐seq analysis combined with experimental validation by Western Blot and immunofluorescence confirmed that *Cs*CA primarily inhibits osteoclast differentiation through the NF‐κB and MAPK signaling pathways.

**Conclusions:**

*Cs*CA inhibits the formation and function of osteoclasts by suppressing the expression of key genes involved in osteoclast differentiation through the inhibition of the NF‐κB and MAPK signaling pathways. This study elucidates the mechanism by which *Cs*CA regulates osteoclasts and suggests its translational potential in preventing and treating hyper‐resorptive bone diseases such as osteoporosis and bone metastasis.

## Graphical abstract

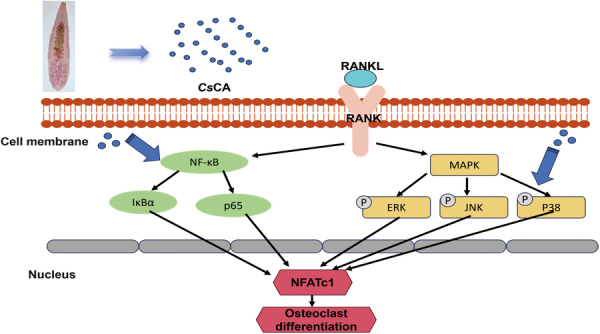

## 1 Introduction

Osteoclastogenesis is a critical process for maintaining bone metabolic homeostasis. Its aberrant activation directly drives pathological bone resorption and serves as a core mechanism underlying skeletal disorders such as osteoporosis and cancer bone metastasis. In osteoporosis, excessive osteoclast activity leads to deterioration of bone microarchitecture and a significant increase in fracture risk; in bone metastasis, it mediates osteolytic destruction, causing severe bone pain, hypercalcemia, and pathological fractures, which considerably compromise patients' quality of life [1]. Osteoclasts originate from precursor cells of the monocyte/macrophage lineage. Their differentiation undergoes a strictly regulated, multi‐step process, encompassing precursor proliferation, differentiation, and ultimate fusion into multinucleated cells [2]. This process is primarily driven by the receptor activator of nuclear factor kappa‐B ligand (RANKL)‐RANK signaling pathway. Activation of this pathway stimulates downstream nuclear factor kappa‐B (NF‐κB) and mitogen‐activated protein kinase (MAPK) pathways, inducing the expression of key transcription factors such as NFATc1 and c‐Fos, which ultimately promote osteoclast differentiation and bone resorption [3, 4]. Given the pivotal role of the RANKL signaling pathway in pathological bone resorption, targeting this pathway has become an important therapeutic strategy for such diseases. Although currently available anti‐resorptive agents (e.g., bisphosphonates or RANKL inhibitors) are effective, long‐term use may be associated with adverse effects or high costs. Therefore, the development of novel treatment strategies capable of precisely modulating osteoclast activity remains of great importance. Among these, targeting the osteoimmune system has emerged as a highly promising new approach. Bone homeostasis and the immune system maintain a dynamic balance through shared cytokines, signaling pathways, and cellular interactions—a field of research known as osteoimmunology. Skeletal homeostasis and the immune system establish a dynamic equilibrium network through shared cytokines, signaling pathways, and cellular interactions, a field termed Osteoimmunology; As the core effector of bone resorption, osteoclast differentiation is regulated by the RANKL/RANK/OPG axis [5, 6, 7] and intimately linked to the functions and cytokine secretion of immune cells including Th17 cells, regulatory T (Treg) cells, and macrophages [8, 9, 10]. Research demonstrates that Th17 cells express high levels of RANKL, binding to RANK on osteoclast precursors to directly induce their development into osteoclasts and accelerate bone loss [11], while simultaneously secreting pro‐inflammatory cytokines such as IL‐17, IL‐21, and interferon‐gamma (IFN‐γ) to exacerbate bone destruction in periodontitis and rheumatoid arthritis [6]; conversely, regulatory T cells (Treg) primarily suppress osteoclast formation through CTLA4‐mediated direct cell‐to‐cell contact, wherein CTLA4 binds B7‐1 and B7‐2 on monocytic osteoclast precursors to inhibit their differentiation [9, 12]. Given this central regulatory role of immune cells in osteoclast differentiation, targeted immunomodulation has emerged as a novel therapeutic strategy for osteolytic bone disorders [13, 14].

In recent years, bioactive molecules derived from parasites have garnered significant attention due to their unique immunomodulatory properties, demonstrating potential regulatory value particularly in the field of osteoimmunology. Taking *Clonorchis sinensis* (*C. sinensis**)*** as an example, its crude antigen (CA) has a role in the anti‐inflammatory function of DC cells by inducing IL‐10 and TGF‐β through activation of extracellular signal‐regulated kinase 1/2 [15]. Bone marrow–derived dendritic cells (BMDCs) treated with *Cs*CA induce the differentiation of naive T helper cells into Th2 cells through the secretion of IL‐10 (rather than IL‐12), significantly enhancing the production of Th2‐type cytokines, such as IL‐4 and IL‐13, both in vitro and in vivo [16]. *C. sinensis* excretory‐secretory products (ESPs) can modulate host immunity through multiple targets: for instance, cysteine protease (CSCP) and miRNAs (e.g., Csi‐let‐7a‐5p) within *C. sinensis* extracellular vesicles (*Cs*EVs) can bidirectionally regulate the NF‐κB signaling pathway, influencing macrophage polarization and inflammatory responses [17, 18]. Furthermore, molecular chaperones from *C. sinensis* (such as rCsHscB) significantly reduce the release of pro‐inflammatory cytokines like TNF‐α and IL‐6 by inhibiting the phosphorylation of the MAPK pathway (ERK, JNK, p38), and restore CD4⁺/CD8⁺ T cell balance in a chronic colitis model, suggesting its potential for systemic immunomodulation [19]. While these immunomodulatory properties of *Cs*CA highlight its potential in inflammatory regulation, a critical gap remains in understanding its direct cellular targets beyond immune cells. Crucially, whether *Cs*CA can directly impact osteoclast precursors to influence bone resorption pathways—independent of immune mediation—remains unexplored. This question holds significant therapeutic implications, as direct targeting of osteoclastogenesis could offer more precise intervention against pathological bone loss. Although the immunomodulatory properties of *Cs*CA highlight its anti‐inflammatory potential, its direct cellular targets beyond immune cells remain unidentified. Notably, whether *Cs*CA can directly regulate osteoclast precursors and bone resorption pathways remains to be investigated. This mechanistic gap carries significant therapeutic implications: direct targeting of osteoclastogenesis may enable precise intervention against pathological bone loss. Current research on *C. sinensis* primarily focuses on carcinogenic mechanisms [20] and immune‐mediated effects (e.g., cytokine modulation), while the direct action of *Cs*CA on osteoclast differentiation has yet to be elucidated. Therefore, this study pioneers systematic investigation into the direct regulatory mechanisms of *Cs*CA on osteoclast differentiation evaluates its therapeutic potential for osteolytic conditions and related disorders, and provides novel strategies for osteoimmunology‐targeted therapies.

## 2 Materials and Methods

### 2.1 *Clonorchis sinensis* Crude Antigen

Adult of *C. sinensis* were collected from the bile ducts of infected rats, selected for structural integrity and viability. The worms were rinsed multiple times with PBS, mechanically fragmented, and resuspended in PBS. The suspension underwent two cycles of freezing at −20°C and thawing. The resulting crude extract was homogenized first with a glass grinder, followed by two rounds of ultrasonic disruption using a cell crusher (Scientz, China) at 30% amplitude, each lasting 15 min under ice‐bath conditions (1 s sonication followed by 2 s rest per cycle). After centrifugation at 13,000 rpm for 20 min at 4°C, the supernatant was collected and stored at −80°C. Protein concentration was quantified with a BCA Protein Assay Kit (Thermo, USA), and all protein preparations were filter‐sterilized through 0.22 μM membranes before cellular assays. The SDS‐PAGE was performed to demonstrate the composition and integrity profile of CsCA (Supporting Information S1: Figure S1).

### 2.2 BMMs Isolation and Osteoclast Culture

Bone Marrow–derived Macrophages (BMMs) were isolated from the femurs and tibias of 4–6‐week‐old male C57BL/6 mice and cultured in complete α‐MEM with 25 ng/mL M‐CSF. Cells were seeded either in 6‐well plate at the density of 1 × 10^5^ cells/well or in 96‐well plates at 6 × 10^3^ cells/well. After cell adhesion, the medium was replaced with complete medium supplemented with 25 ng/mL M‐CSF and 50 ng/mL RANKL. Osteoclast differentiation was induced following two rounds of medium change and RANKL stimulation. Terminal differentiation was assessed by Tartrate‐Resistant Acid Phosphatase (TRAcP) staining or total RNA extraction for analysis.

### 2.3 TRAcP Staining Assay

BMMs were harvested from 4 to 6‐week‐old male C57BL/6 mice and cultured in 96‐well plates to induce osteoclast differentiation. Following osteoclast formation, the cells were washed once with 1× PBS and fixed with 4% (v/v) paraformaldehyde. Osteoclasts were subsequently identified by staining with the TRAcP staining solution. TRAcP‐positive multinucleated cells containing three or more nuclei were defined as osteoclast‐like (OCL) cells.

### 2.4 RNA Isolation and Complementary DNA (cDNA) Synthesis

BMMs cultured in 6 well plate to generate osteoclasts, total RNA extracted using ultra‐fast single‐column animal cell Total RNA extraction kit (vazyme, China). After discarding the supernatant of cell culture medium, lyse in a culture dish, add 500 μL Buffer CRL to each well to fully cover the cell surface, and then blow the cells repeatedly with a pipette to make them fall off; transfer all the above lysates to RNA Columns I, centrifuge at 12,000 rpm for 30 s, and discard the waste liquid. Add 500 μL of Buffer RWA (anhydrous ethanol has been added) to RNA Columns I, centrifuge at 12,000 rpm for 30 s, and discard the waste liquid. Add 500 μL Buffer RW (absolute ethanol has been added) to RNA Columns I, centrifuge at 12,000 rpm for 1 min, and discard the waste. RNA Columns I were returned to the collection tube and centrifuged at 12,000 rpm for 1 min to prevent alcohol contamination. RNA Columns I was carefully transferred to a new 1.5 mL RNase‐free centrifuge tube, and 40 μL of RNase‐free ddH_2_O was dropped into the center of the adsorption column membrane. RNA was incubated at room temperature for 1 min, centrifuged at 12,000 rpm for 1 min to elute RNA, and RNA concentration and purity were determined. Reverse transcribe RNA into cDNA. Prepare enzyme‐free 100 μL centrifuge tubes, add 1 μg RNA and 2 μL 8 × gDNA Eraser Premix to each tube according to reagent instructions, supplement RNase Free H_2_O water to 16 μL, blow and mix well, 42°C for 2 min. Then add 4 μL of 5 × RT Premix to each tube, mix well by blowing, reverse transcription reaction, 37°C for 10 min, 85°C for 5 s, and store at −20°C.

### 2.5 Quantitative PCR

cDNAs was isolated as described above, and 20 μL of reaction system per well was prepared according to reagent instructions: 0.4 μL primer (F), 0.4 μL primer (R), 10 μL 2 × ChamQ Universal SYBR qPCR Master Mix, 1 μL cDNA (diluted 10‐fold), 7.2 μL DEPC water. In a real‐time fluorescence quantitative PCR instrument (QuantStudio 3, USA), the program was set according to the instructions: 94°C for 10 min, followed by 45 cycles at 95°C × 15 s and 60°C × 60 s. All data were normalized to β‐actin using a comparison threshold cycle. The primers utilized in the current investigation are presented in Table 1.

**Table 1:** Primer sequences of osteoclast‐specific expressed genes.

Target gene	Primer sequence (5ʹ–3ʹ)
***Nfatc1***	Forward:GGTGCTGTCTGGCCATAACT
	Reverse:GAAACGCTGGTACTGGCTTC
***Ctsk***	Forward:AGGCGGCTCTATATGACCACTG
	Reverse:TCTTCAGGGCTTTCTCGTTC
***C‐fos***	Forward:CCAGTCAAGAGCATCAGCAA
	Reverse:AAGTAGTGCAGCCCGGAGTA
***Atp6v0d2***	Forward:GTCCCATTCTTGAGTTTGAGG
	Reverse:ATCTCCTTCTGCATCCTGTC
***Dcstamp***	Forward:TCTGCTGTATCGGCTCATCTC
	Reverse:ACTCCTTGGGTTCCTTGCTT
***Mmp‐9***	Forward:GAAGGCAAACCCTGTGTGTT
	Reverse:AGAGTACTGCTTGCCCAGGA
***ACP5***	Forward:ACGGCTACTTGCGGTTTCA
	Reverse:TCCTTGGGAGGCTGGTCTT
***Gapdh***	Forward:TCCTCCCTGGAGAAGAGCTA
	Reverse:ACACATTGGGGGTAGGAACA

### 2.6 Western Blot Assay

BMMs were cultured in 6‐well plates and treated differently depending on the experimental results. Total protein was extracted using ice‐cold lysis buffer containing phosphatase and protease inhibitors, followed by incubation on ice for 30 min. The lysate was centrifuged at 12,000 rpm for 15 min at 4°C to collect the supernatant. Protein concentration was determined using a BCA assay kit (Thermo Scientific). Proteins were denatured in loading buffer by boiling for 10 min, separated by SDS‐PAGE, and transferred onto PVDF membranes via the wet transfer method (300 mA, 1.5 h, ice bath). After blocking with rapid blocking buffer (Epizyme, China) for 10 min, the membranes were incubated overnight at 4°C with the following primary antibodies: NFATc1 (1:200, Santa Cruz Biotechnology, cat. no. Sc 7294), c‐Fos (1:1000, Abcam, cat. no. ab222699), CTSK (1:200, Santa Cruz Biotechnology, cat. no. sc48353), p‐ERK1/2 (1:1000, Cell Signaling Technology, cat. no. 4695), ERK1/2 (1:1000, Cell Signaling Technology, cat. no. 4695), p‐p38 (1:1000, Cell Signaling Technology, cat. no. 4511), p38 (1:1000, Cell Signaling Technology, cat. no. 8690), p‐JNK (1:1000, Cell Signaling Technology, cat. no. 4668), JNK (1:1000, Cell Signaling Technology, cat. no. 4668), IκBα (1:1000, Cell Signaling Technology, cat. no. 4812), and β‐actin (1:10000, Proteintech, cat. no. 66009‐1‐Ig). Subsequently, the membranes were incubated at room temperature for 1 h with HRP‐conjugated secondary antibodies (1:10,000 dilution, Huabio, China): either goat anti‐rabbit IgG (cat. no. HA1001) or goat anti‐mouse IgG (cat. no. HA1006), as appropriate. After thorough washing with TBST, protein bands were visualized using an ECL substrate and imaged with an iBright imaging system (Invitrogen, USA). Band intensities were quantified using ImageJ software.

### 2.7 RNA Sequencing (RNA‐Seq)

BMMs were cultured in six‐well plates. Cells were divided into PC group (50 ng/mL RANKL) and *Cs*CA group (50 ng/mL RANKL+*Cs*CA 40 μg/mL). After 72 h treatment, the complete culture medium was aspirated and washed three times with pre‐cooled 1× PBS. Add 1 mL Trizol into each well, blow repeatedly for 5 times and transfer to 1.5 mL EP tube, then quickly put it into liquid nitrogen for quick freezing, and then send it to HiploX Technology Co. Ltd. for transcriptome sequencing by dry ice: extract RNA sample and detect its quality; transcribe RNA template into cDNA after qualified sample, and construct sample library after cDNA fragmentation; After the library is successfully constructed, the library is tested. After the library inspection is qualified, different libraries are pooled according to the effective concentration and the demand of target off‐machine data volume, and then Illumina PE150 sequencing is performed.

### 2.8 p65 Nuclear Translocation Immunofluorescence Assay

BMMs were plated and maintained in complete medium supplemented with 25 ng/mL M‐CSF at 37°C in a 5% CO₂ atmosphere until full adhesion was achieved. On the following day, the culture medium was exchanged for serum‐free α‐MEM to subject the cells to serum deprivation for 1 h. Subsequently, the cells were exposed to either CsCA or vehicle control for 1 h, followed by stimulation with RANKL for 30 min. After treatment, the medium was removed and the cells were fixed with 4% paraformaldehyde for 10 min, rinsed thoroughly three times with PBS, and permeabilized using 0.1% Triton X‐100 for 5 min. Nonspecific binding sites were blocked with 3% BSA for 1 h at room temperature. The cells were then incubated overnight at 4°C with a primary antibody specific to p65 (1:100 dilution, Cell Signaling Technology, cat. no. 8242). After removal of the primary antibody, a Cy5‐conjugated secondary antibody was applied and incubated for 1 h at room temperature. Nuclei were labeled with DAPI for 5 min before fluorescence microscopic visualization and image acquisition.

## 3 Results

### 3.1 *Cs*CA Suppressed RANKL‐Induced Osteoclast Differentiation In Vitro

**Figure 1:**
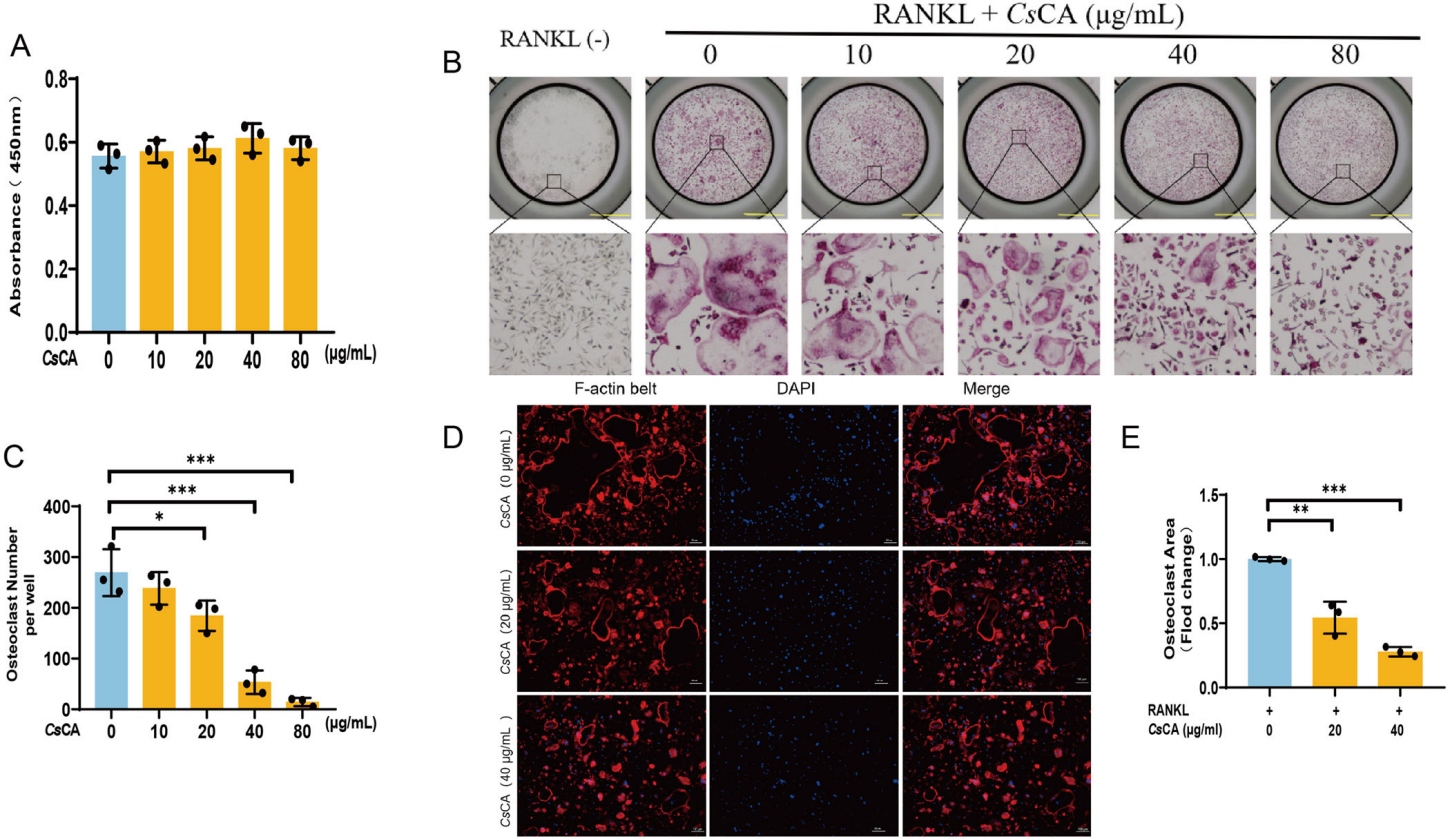
*Cs*CA inhibited RANKL‐induced osteoclastogenesis in vitro. (A) The results of cytotoxicity. CCK‐8 assay showed no significant toxic effect at doses below 80 μg/mL (*n* = 3/group). (B) TRAcP staining after *Cs*CA intervention. *Cs*CA dose‐dependently inhibited osteoclastic differentiation of BMMs. Scale bar = 2000 μM (C) Quantification of TRAcP staining. Cells were defined as osteoclasts when they had ≥ 3 nuclei (*n* = 3/group). (D) Osteoclast actin ring (F‐actin belt) staining. Scale bar = 100 μM. (E) Quantification of actin belt area was performed using ImageJ software (*n* = 3/group). **p < 0.01, ***p < 0.001.

Cytotoxicity assessment using CCK‐8 assay indicated that *Cs*CA did not impair the proliferation of BMMs after 48 h of treatment at concentrations ranging from 0 to 80 μg/mL (Figure 1A), suggesting its non‐cytotoxic nature toward osteoclast precursors. Furthermore, following 5–7 days of culture, *Cs*CA was found to inhibit osteoclast differentiation in a dose‐dependent manner, with the most significant reduction in osteoclast number observed at 80 μg/mL (Figure 1B,C). Additionally, *Cs*CA treatment disrupted the formation of F‐actin rings, which play a critical role in osteoclast maturation and resorptive function. Using fluorescence microscopy, we examined F‐actin ring formation during RANKL‐induced differentiation of BMMs into mature osteoclasts. Control osteoclasts displayed well‐defined F‐actin ring structures, whereas cells treated with 20 or 40 μg/mL *Cs*CA exhibited a marked reduction in F‐actin ring size. The inhibitory effect was more substantial at 40 μg/mL (Figure 1D,E).

### 3.2 *Cs*CA Downregulated the Expression of Osteoclast‐Specific Genes and Proteins During Osteoclast Differentiation

**Figure 2:**
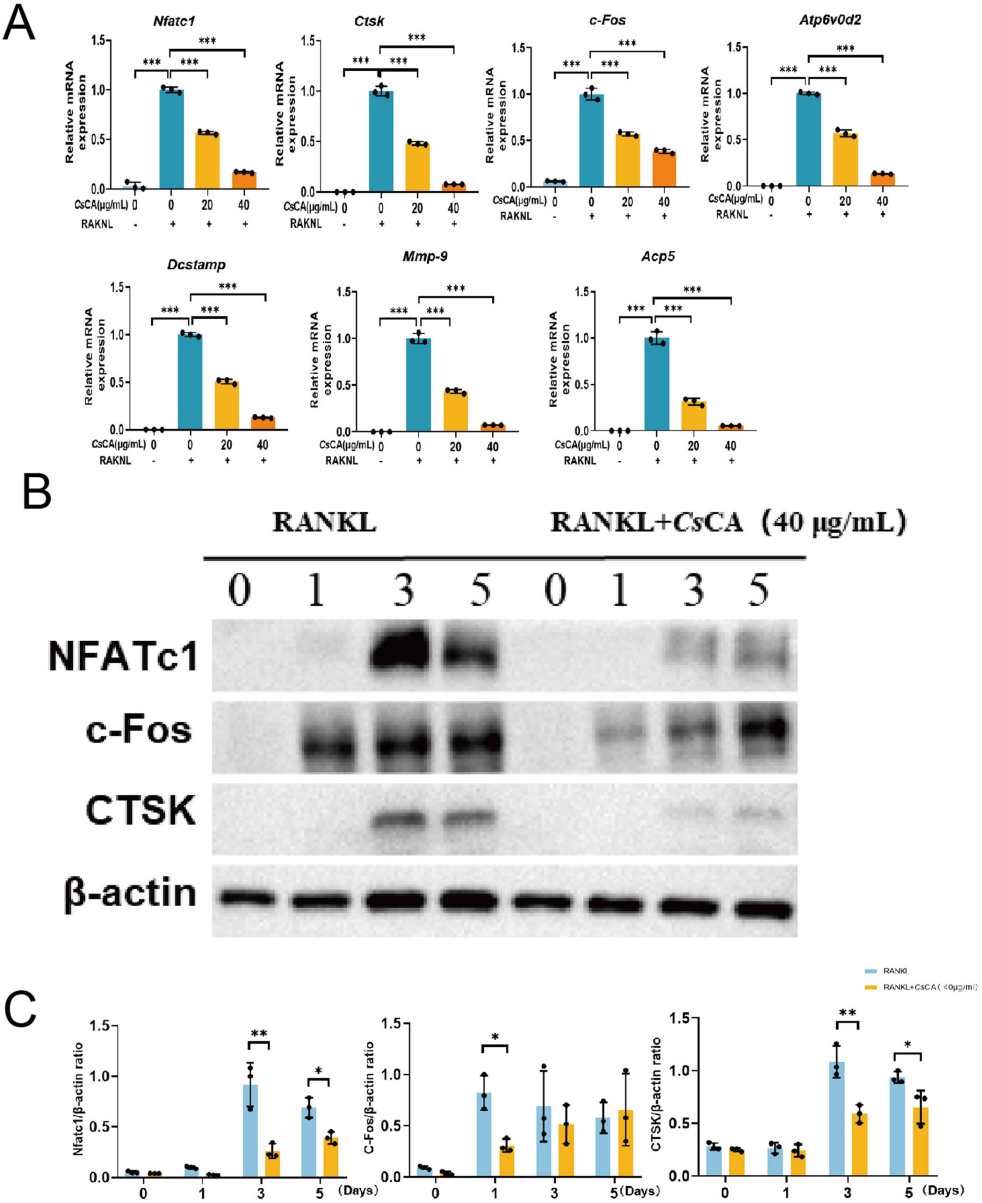
*Cs*CA downregulated the expression of osteoclast‐specific markers. (A) *Cs*CA induced concentration‐dependent suppression of mRNA expression of osteoclast‐related genes, including *Nfatc1, Ctsk, c‐Fos*, *Atp6v0d2, Dc‐stamp, Mmp‐9* and *Acp5* (*n* = 3/group). (B) Western blot analysis revealed that *Cs*CA markedly suppressed the protein expression of NFATc1, c‐Fos, and CTSK at Day 3 and Day 5 of osteoclast differentiation. (C) Western blot band intensities were quantified with ImageJ (*n* = 3/group). *p < 0.05, **p < 0.01, ***p < 0.001.

Quantitative real‐time PCR analysis demonstrated that *Cs*CA significantly suppressed the mRNA expression of key osteoclast‐related genes, including Nfatc1, Ctsk, c‐Fos, Atp6v0d2, Dc‐stamp, Mmp9, and Acp5 (Figure 2A). Consistent with the transcriptional changes, Western blot analysis revealed a concomitant reduction in the protein levels of NFATc1, c‐Fos, and CTSK following *Cs*CA treatment (Figure 2B,C). These results collectively indicate that *Cs*CA attenuates osteoclast differentiation and function through the suppression of osteoclast‐specific genes and proteins.

### 3.3 Transcriptomic Analysis of the Molecular Mechanisms by Which *Cs*CA Regulated Osteoclast Differentiation

**Figure 3:**
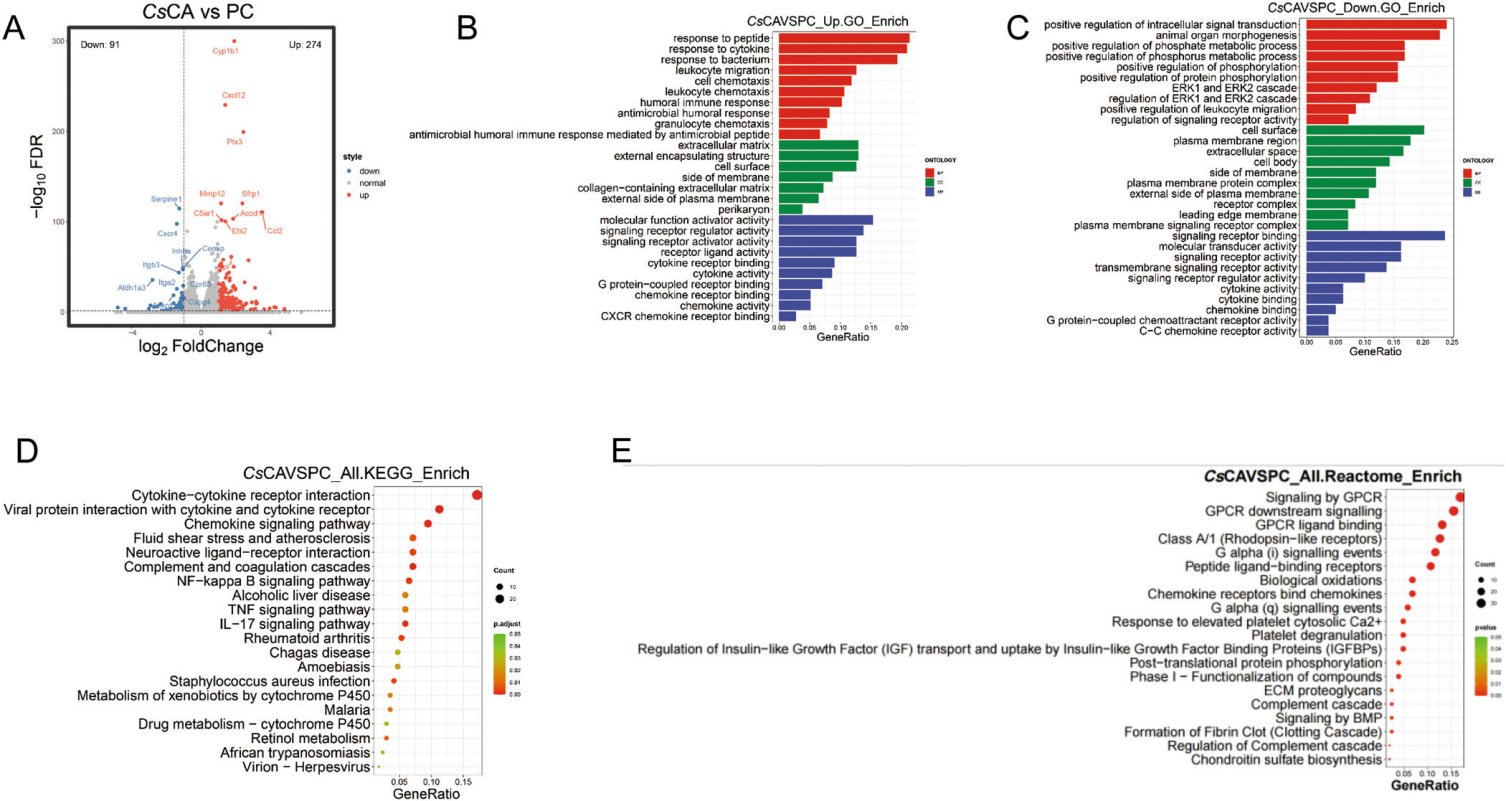
Distinct RNA profiles between *Cs*CA and PC group. (*n* = 3/group) (A) Volcano diagram of DEGs between *Cs*CA and PC group. Enrichment analysis of GO terms for up (B) and down (C) regulated genes between *Cs*CA and PC group. (D) KEGG analysis of regulated genes between *Cs*CA and PC group. (E) Reactome pathway Enrichment Analysis of DEGs between *Cs*CA and PC group.

To elucidate the mechanism by which *Cs*CA regulates osteoclast differentiation, transcriptomic analysis was conducted by treating cells with *Cs*CA during osteoclast differentiation. A total of 365 differentially expressed genes (DEGs) were identified, including 274 upregulated genes and 91 downregulated genes between *Cs*CA and PC group (screening criteria: fold change *FC* ≥ 2, *p* < 0.05; Figure 3A). The GO analysis revealed that the upregulated genes were predominantly enriched in categories such as cytokines, extracellular matrix, signaling receptors, and cytokine activity (Figure 3B), and downregulated genes are primarily enriched in positive regulation of intracellular signal transduction, positive regulation of phosphorylation, cell surface, plasma membrane region and molecular transducer activity, etc (Figure 3C). The KEGG analysis revealed that genes were significantly enriched in Cytokine–cytokine receptor interaction, Chemokine signaling pathway, NF–kappa B signaling pathway, and so forth (Figure 3D). Reactome pathway enrichment analysis revealed that the DEGs were predominantly enriched in GPCR signaling, complement‐ and chemokine‐mediated immune responses, and metabolic regulation pathways, and so forth (Figure 3E).

### 3.4 *Cs*CA Suppressed RANKL‐Induced Activation MAPK and NF‐κB Signaling Pathways

**Figure 4:**
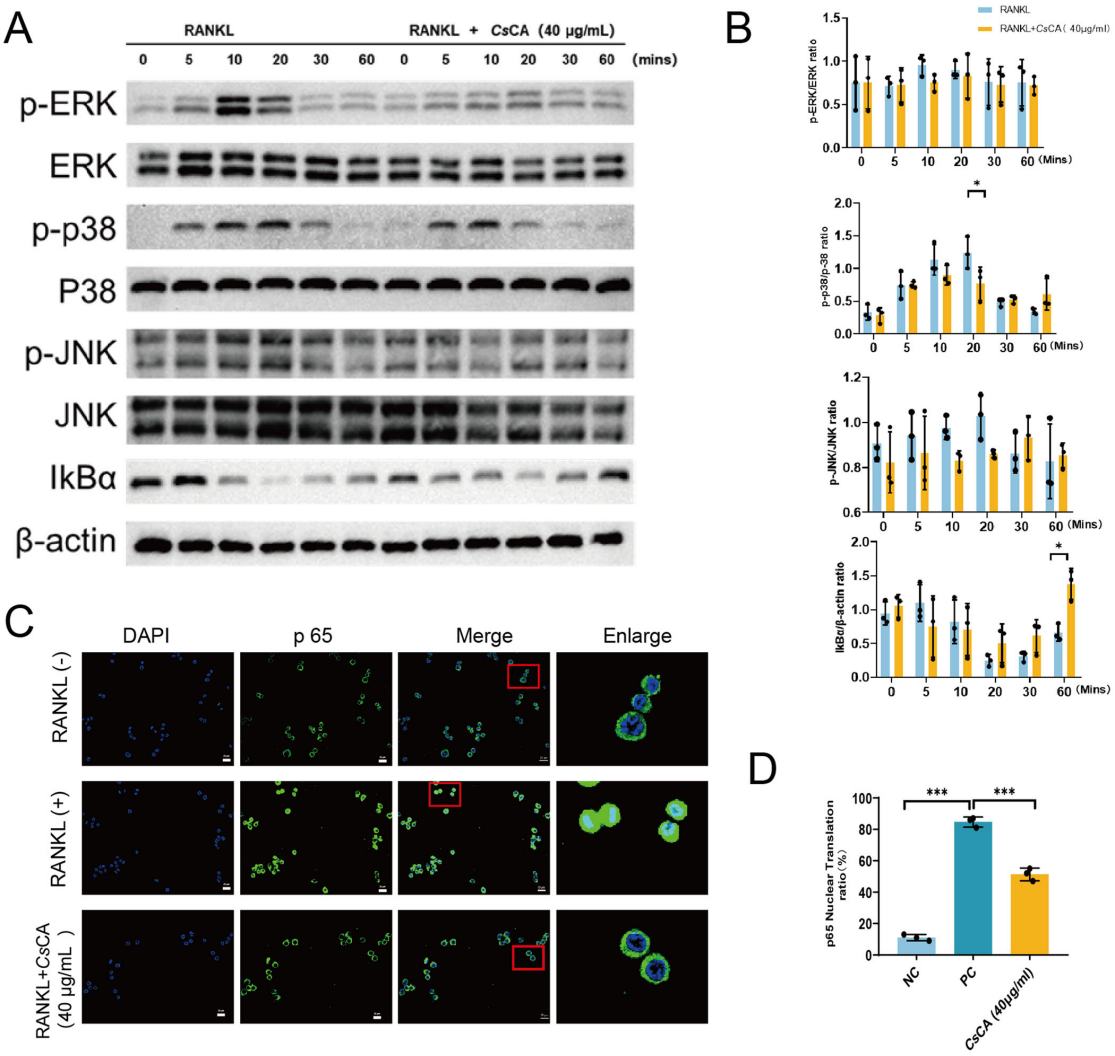
Effect of *Cs*CA on RANKL‐induced MAPK and NF‐κB signaling pathways. (A) *C* Representative Western blots demonstrating that CsCA attenuates RANKL‐stimulated phosphorylation of p38 at 20 min and inhibits IκB‐α degradation at 60 min. (B) Western blot band intensity was performed using ImageJ software (*n* = 3/group). (C) Fluorescence images illustrating nuclear translocation of p65 (green) following RANKL stimulation, with nuclei stained by DAPI (blue). Scale bar = 20 µM. (D) Quantification of p65 nuclear translocation ratio (*n* = 3/group). *p < 0.05, **p < 0.01, ***p < 0.001.

The above analysis found that *Cs*CA could be the main function and the inflammatory pathway to regulate osteoclast differentiation. It is well‐known that osteoclast differentiation and maturation processess are intricately associated with MAPK and NF‐κB signaling pathways. In this study, BMMs were pretreated with 40 μg/mL *Cs*CA for 1 h in 6‐well plates and subsequently stimulated with RANKL (50 ng/mL). Western Blot analysis revealed that *Cs*CA significantly inhibited RANKL‐induced phosphorylation of p38 at the 20‐min time point. Additionally, *Cs*CA markedly attenuated the degradation of IκB‐α at 60 min poststimulation, thereby suppressing the activation of the NF‐κB pathway (Figure 4A,B). These results were corroborated by fluorescence microscopy, which showed that *Cs*CA inhibited the nuclear translocation of p65 (green), with nuclei stained with DAPI (blue) (Figure 4C,D).

## 4 Discussion

Numerous parasitic infections (particularly helminths) and their derivatives, such as excretory/secretory products, have been demonstrated to possess immunomodulatory and immunosuppressive potential. Experimental infection with *Schistosoma japonicum*, *S. mansoni*, *Ascaris suum*, or *Hymenolepis diminuta*, or the use of their protein products, has been shown to inhibit collagen‐induced arthritis in mice [21, 22, 23, 24, 25]. These findings suggest that parasite‐derived antigens may influence bone‐related pathological processes through modulation of the immune system. Against this background, the present study focused on the direct regulatory effects of *Cs*CA on osteoclast differentiation. The experiments in this study showed that *Cs*CA exhibits no significant toxicity toward bone marrow–derived macrophages (BMMs) within the 0–80 μg/mL concentration range but dose‐dependently inhibits RANKL‐induced osteoclast differentiation. TRACP staining revealed that *Cs*CA concentrations > 20 μg/mL significantly reduced the number of multinucleated osteoclasts. Furthermore, fluorescence staining (rhodamine‐conjugated phalloidin) showed that *Cs*CA suppressed the formation of characteristic actin rings and nuclear fusion in osteoclasts, suggest that *Cs*CA may exert an osteoprotective effect by interfering with osteoclast function. It is the first exposed that *Cs*CA can inhibit osteoclast differentiation.

This study revealed that *Cs*CA exerts inhibitory effects by synergistically suppressing both the NF‐κB and MAPK signaling pathways. Specifically, *Cs*CA treatment significantly delayed I‐κBα degradation and blocked nuclear translocation of p65. It also inhibited phosphorylation of ERK, JNK, and p38, with the suppression of p38 reaching statistical significance (*p* < 0.05) as early as 20 min after RANKL stimulation. These findings suggest that *Cs*CA may preferentially target the p38–MAPK signaling axis, thereby interfering with the activation of the NFATc1/c‐Fos transcriptional complex essential for osteoclast differentiation. In contrast to other parasite antigens reported in the literature—such as those from *Schistosoma mansoni* and *Trichinella spiralis*, which primarily alleviate arthritis by upregulating regulatory T cells (FoxP3⁺ Tregs) and coordinating anti‐inflammatory cytokine networks to indirectly modulate immune responses tokines [24]—*Cs*CA acts directly on osteoclast precursors and inhibits RANKL downstream signal transduction, demonstrating unique multi‐target osteoprotective properties. Notably, *Cs*CA exhibits functionally opposite effects to hydatid antigen B (Hyd‐B): while recombinant Hyd‐B as a single protein promotes osteoclast differentiation by enhancing ERK/NF‐κB signaling via the TAZ‐TAK1 axis [26], *Cs*CA inhibits osteoclast formation and exerts bone protection through broad suppression of key osteoclastogenic signaling pathways. This functional discrepancy not only originates from differences in their antigen composition and biological characteristics of the source parasite, but also reflects their distinct roles in host–parasite interactions: Hyd‐B is primarily associated with bone destruction in cystic echinococcosis, whereas *Cs*CA may influence bone metabolism under chronic infection conditions via multi‐target immunomodulatory mechanisms. Another noteworthy phenomenon is that although *Cs*CA demonstrates clear inhibitory effects on osteoclast differentiation, *C. sinensis* infection itself has been reported to exacerbate bone destruction in arthritis models [27]. This seemingly paradoxical phenomenon arises from fundamental differences between the process of infection and purified antigens: as a crude antigen mixture, CsCA tends to induce anti‐inflammatory or regulatory immune responses in a simplified system, thereby directly inhibiting the RANKL signaling pathway. In contrast, live worm infection is a continuous and dynamic process in which the parasites not only constantly release excretory‐secretory products (ESPs) and extracellular vesicles (EVs)—containing immunomodulatory molecules such as miRNAs that may persistently activate certain inflammatory pathways—but may also cause mechanical damage and accumulation of metabolic byproducts due to larval migration, collectively triggering intense local and systemic inflammatory responses. This observation is consistent with previous studies indicate therapeutic potential of *C. sinensis*‐derived proteins exhibit anti‐inflammatory and therapeutic potential in ankylosing spondylitis [28] and DSS‐induced colitis in mice [29], highlighting the significant immunomodulatory capacity of *C. sinensis* components and underscoring the high complexity of parasite‐host interactions.

This study is the first to reveal that *Cs*CA inhibits osteoclast differentiation through a dual‐targeting mechanism, specifically by suppressing both the MAPK and NF‐κB signaling axes. This unique “immune‐bone metabolism” regulatory function underscores its therapeutic potential for treating bone‐destructive diseases. Furthermore, our findings provide novel insights into parasite‐host interactions and reveal opportunities for developing targeted interventional strategies against osteolytic disorders and inflammatory arthritis.

## 5 Study Limitations

This study has several limitations. Firstly, although we utilized crude *Cs*CA, the specific bioactive protein(s) responsible for the observed inhibitory effects on osteoclastogenesis remain unidentified. The complex composition of the crude antigen means that the exact molecular mechanism could not be definitively elucidated. Secondly, our findings are primarily based on in vitro models using bone marrow–derived macrophages. The lack of complementary in vivo experiments limits our ability to fully assess the physiological relevance and therapeutic potential of *Cs*CA in the host. Future studies focusing on fractionating the crude antigen to identify the key functional components, coupled with validation in animal models of bone loss, would be essential to strengthen our conclusions and provide deeper mechanistic insights.

## 6 Conclusions

Our study demonstrated that *Clonorchis sinensis* crude antigen (*Cs*CA) inhibits osteoclast differentiation through dual suppression of the NF‐κB pathway (via IκBα stabilization and impairment of p65 nuclear translocation) and the MAPK pathway (via inhibition of p38 phosphorylation). These findings highlight the potential of *Cs*CA as a novel therapeutic candidate for mitigating osteolytic bone diseases.

## Author Contributions

**Lili Tang** and **Shanshan He:** validation, investigation, methodology, supervision, writing – review and editing, data curation. **Buguo Ma:** methodology, writing – review and editing. **Zeli Tang:** methodology, validation. **Tingzheng Zhan** and **Jinmin Zhao:** supervision, formal analysis, visualization. **Suyu Xiao** and **Jilong Wang:** methodology, validation, formal analysis, visualization. **Jiake Xu** and **Yunliang Shi:** conceptualization, methodology, writing – original draft, writing – review and editing, investigation, supervision, project administration, data curation.

## Ethics Statement

All animal procedures were performed in accordance with relevant legal and ethical standards following a protocol (no. 202403039) approved by the Animal Care and Welfare Committee of Guangxi Medical University (Nanning, China), and conducted in compliance with the ARRIVE guidelines.

## Conflicts of Interest

The authors declare no conflicts of interest.

## Supporting information


**Figure S1**. Adult worms of *Clonorchis sinensis* and *Cs*CA protein. (A) Adult *Clonorchis sinensis*. (B) Silver staining was performed to detect *Cs*CA protein.
