# Supplementary figures and images for: Clonorchis sinensis Crude Antigen Suppresses Osteoclast Differentiation via Modulation of the NF‐κB and MAPK Signaling Pathway
Source: Immun Inflamm Dis. 2025 Nov 5;13(11):e70292. doi: 10.1002/iid3.70292 (PMC12589821; doi:10.1002/iid3.70292)

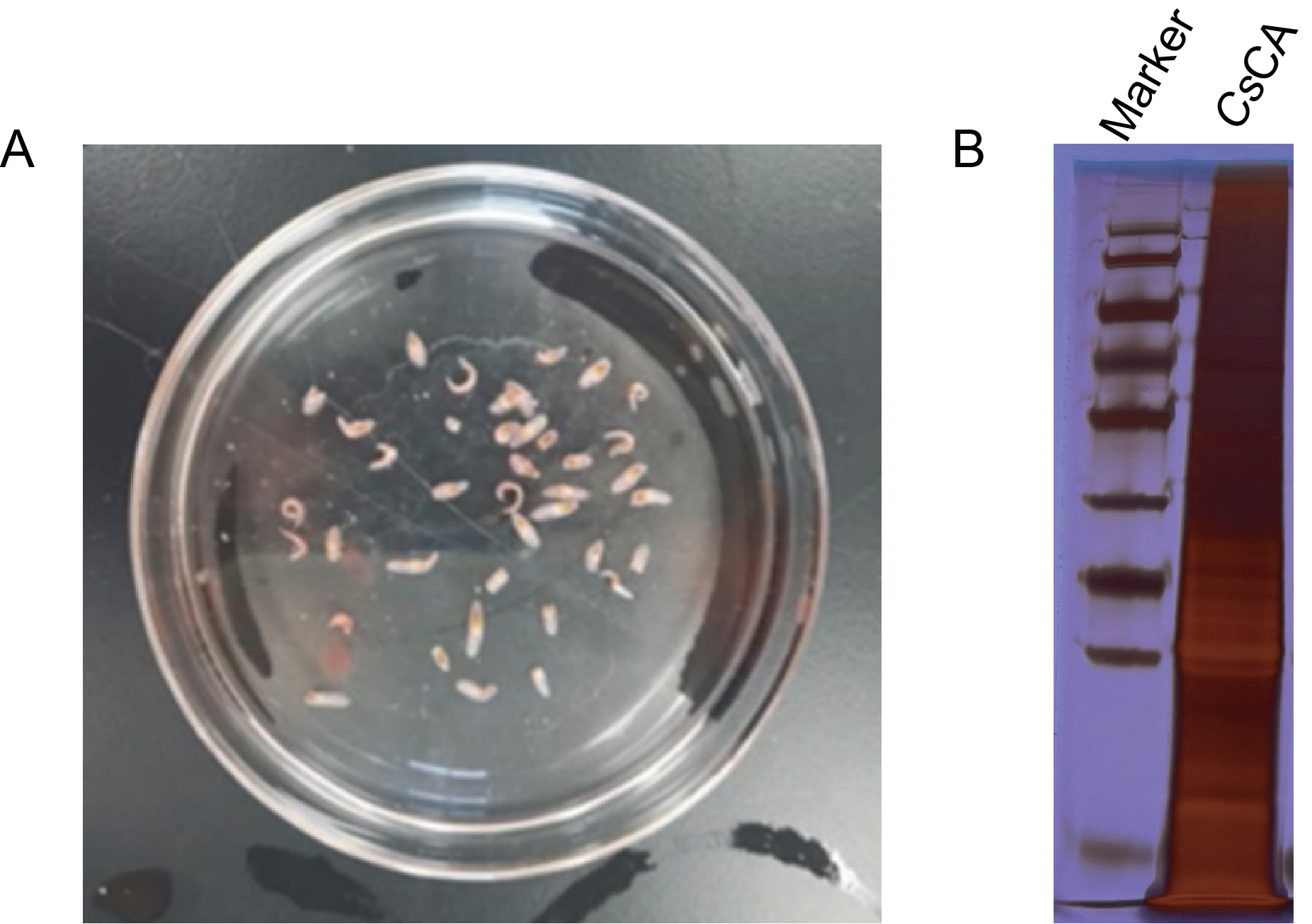

Supplement: Supplementary file 1 — Figure S1. Adult worms of Clonorchis sinensis and CsCA protein. (A) Adult Clonorchis sinensis. (B) Silver staining was performed to detect CsCA protein. [file IID3-13-e70292-s001.jpg]
